# Asthma in the elderly: a study of the role of vitamin D

**DOI:** 10.1186/1710-1492-10-48

**Published:** 2014-09-05

**Authors:** Michele Columbo, Reynold A Panettieri, Albert S Rohr

**Affiliations:** Allergy and Immunology Division, Bryn Mawr Hospital, Bryn Mawr, PA USA; Pulmonary, Allergy and Critical Care Division, University of Pennsylvania School of Medicine, Philadelphia, PA USA

**Keywords:** Asthma, Vitamin D, Elderly

## Abstract

**Background:**

Asthma in the elderly is poorly understood and vitamin D deficiency and insufficiency are very common in older individuals. We studied the role of vitamin D in elderly asthmatics.

**Methods:**

Asthmatics subjects, age 65 and older, were followed every 4 weeks for 12 weeks in the late fall and winter. During the study period they took 2,000 I.U. vitamin D3 daily. Serum 25-Hydroxyvitamin D and calcium were measured at baseline and study end.

**Results:**

Twenty nine percent of subjects were deficient and 50% insufficient in serum vitamin D at baseline. Serum vitamin D increased from 24.3±9.2 ng/ml (60.7±23 nmol/L) to 34±7.1 ng/ml (84.9±17.7 nmol/L) at the end of the study (p<0.001), whereas calcium was unchanged. We found no significant association between vitamin D and subjects' demographics. Vitamin D was similar in men and women. There was no association between serum vitamin D and inhaled steroid dose. Vitamin D was significantly lower in subjects with uncontrolled asthma (Asthma Control Test, ACT≤19) compared to the ones with well controlled symptoms (p<0.05). In subjects with uncontrolled asthma at baseline, ACT scores increased significantly at the end of the study (p<0.04), but not at 4 and 8 weeks. Spirometric values remained unchanged throughout the study.

**Conclusions:**

Elderly asthmatics very commonly have vitamin D deficiency or insufficiency. Serum vitamin D levels were lower in subjects with uncontrolled asthma. In these subjects, vitamin D supplementation for 12 weeks led to improved ACT scores. Larger, randomized, placebo controlled studies are required to further evaluate whether vitamin D supplementation may improve asthma symptoms in this population.

**Trial registration:**

ClinicalTrials.gov NCT01730976.

## Findings

### Background

About 13% of the U.S. population is older than 65 years and this percentage will nearly double by the year 2050 [1]. Approximately 7% of individuals 65 years old and older have asthma [1]. However, little is known about asthma in this group of patients as most studies have not included elderly subjects. Elderly asthmatics are more likely to be underdiagnosed, undertreated, and hospitalized when compared to younger asthmatics [1]. Asthma’s pathophysiology is different in the elderly and there are many challenges in the treatments of these patients [1]. We have recently shown that measuring exhaled nitric oxide in elderly asthmatics may be unwarranted [2].

Several studies have suggested that vitamin D deficiency and insufficiency are extremely common, even in people with abundant sun exposure [3, 4]. The vitamin D receptor is present in the bronchial smooth muscle [5] and lower vitamin D levels may lead to bronchial smooth muscle proliferation, cytokine release and airway remodeling [5–8]. In addition, vitamin D has been shown to play a role in immunomodulation by interacting with T lymphocytes, dendritic cells, mast cells, monocytes and macrophages [9]. Therefore, it is not surprising that vitamin D deficiency has been associated with airway hyperresponsiveness, lower pulmonary function, and worse asthma control [3, 10].

Older age and vitamin D deficiency are predictors of higher all cause mortality [11]. We are unaware of studies that have investigated the role of vitamin D and its supplementation in elderly asthmatics, a group particularly at risk for vitamin D insufficiency and deficiency.

Therefore, we performed a pilot study in elderly asthmatics investigating whether any association exists between serum vitamin D levels and age, Body Mass Index (BMI), and disease duration. We also studied whether serum vitamin D levels vary in relationship to asthma drugs, spirometric values and at different levels of asthma control, measured by the ACT. Finally, we looked at the effect of vitamin D3 supplementation (2,000 I.U./day) for 12 weeks on ACT scores and spirometric measurements. As sun exposure is the main source of vitamin D, the results of studies of vitamin D and its supplementation can be affected by seasonal fluctuations in sun exposure. For that reason our study was performed in the late fall and winter.

### Methods

This study was performed in the period between November 1, 2012 and March 18, 2013. Twenty-eight subjects 65 years old and older with asthma (25 white, 3 African-American) followed in an Allergy and Immunology practice in suburban Philadelphia were included in the study. Current smokers or subjects with more than 10 pack-years history of smoking were excluded. Almost all study subjects were lifetime nonsmokers. Subjects with hypercalcemia (≥10.5 mg/dl), with hypervitaminosis D (≥100 ng/ml), those taking digoxin or with liver disease were also excluded. None of the study subjects had other comorbidities such as renal disease, depression, or collagen vascular disease. Additional vitamin D containing supplements were not allowed for at least two weeks prior to the initial study visit and for the duration of the study. This study was approved by the Main Line Hospitals Institutional Review Board.

The presence of atopy was verified by allergy skin tests for relevant environmental allergens. Spirometric values were obtained according to the ATS/ERS guidelines by a KoKo Spirometer (nSpire Health, Inc, Longmont, Colorado). 25-Hydroxy vitamin D and calcium were measured in the serum of the study subjects at the Main Line Health Laboratories, Bryn Mawr, PA. Subjects with serum vitamin D levels of less than 20 ng/ml (49.9 nmol/L) were classified as deficient, whereas levels ranging between 20 and 29 ng/ml (49.9 and 72.4 nmol/L) were considered as vitamin D insufficiency. Serum vitamin D and calcium levels were measured at study entry and at the end of the study (12 weeks later).

The inhaled steroids used by the study subjects were fluticasone (15), budesonide (6), mometasone (3), and beclomethasone (1). Steroid doses were expressed as fluticasone-equivalent. The long-acting bronchodilators were salmeterol (12) and formoterol (7). Montelukast was the only leukotriene antagonist used by the study subjects.

The study subjects were followed for 12 weeks with evaluations including a brief history, physical exam, collection of ACT scores and performance of a spirometry at baseline and at 4, 8, and 12 weeks. The ACT is a commonly used tool that allows patients to grade their asthmatic symptoms. Patients report their asthmatic symptoms during the previous four weeks on a scale from 1 (severe) to 5 (no symptoms) by answering five questions about their asthma. Values over 19 are considered as indicative of good asthma control.

Each study subject was instructed to take one softgel of Vitamin D3 2,000 IU (Vitamin Shoppe, North Bergen, NJ) daily every morning for 12 weeks. The study subjects were asked to bring the bottle containing vitamin D3 softgels at the three additional study visits for a visual inspection. The bottles and the unused softgels were collected at the end of the study and the remaining softgels counted and their number recorded.

Data were analyzed by the two tailed, unpaired Student’s t test and the Pearson correlation coefficient. Descriptive variables were expressed as means and standard deviations. Significance was accepted at alpha = .05 with no adjustment for multiple comparisons.

### Results

Table 1 shows the study subjects’ characteristics at baseline. Most subjects were atopic (93%), on inhaled steroids (89%), had well controlled asthma (79%) and coexistent rhinitis (89%). Vitamin D deficiency and insufficiency were very common. In particular, 29% of subjects were deficient (<20 ng/ml) and 50% insufficient (20-29 ng/ml) in serum vitamin D at baseline.

**Table 1:** **Subjects’ characteristics at baseline**

Age (years, range)	72.6 ± 5.8, 65-84
Sex (F/M)	16/12
BMI	25 ± 3.2
Atopy	25/28
Duration of asthma (years)	34.3 ± 21.1
Rhinitis	25/28
Gastroesophageal reflux disease	7/28
Osteoporosis	2/28
Heart disease	2/28
Inhaled steroids (dose, range)	25/28(470 ± 341, 0-1200 mcg/day)
Long-acting bronchodilators	19/28
Leukotriene antagonist	13/28
ACT score	22.4 ± 3.2
Serum vitamin D (ng/ml)	24.3 ± 9.2
Serum calcium (mg/dl)	9.6 ± 0.3

We found no significant association between serum vitamin D and age, BMI, and duration of asthma (Table 2). Vitamin D was similar in men and women (24.4 ± 10.3 vs. 24.1 ± 8.1 ng/ml, respectively).

**Table 2:** **Associations between subjects’ serum vitamin D and demographics**

	Correlation coefficient (r)	P value
Age	-0.02	0.92
BMI	-0.24	0.22
Duration of asthma	0.25	0.2

At baseline, serum vitamin D was similar in subjects using higher doses of inhaled steroids (>400 mcg/day, n = 17) when compared to the ones on lower doses (Table 3). Inhaled steroid dose was not associated with serum vitamin D (r = -0.2, p = 0.31). There was no significant difference in serum vitamin D in subjects on long-acting bronchodilators (26 ± 9.8 ng/ml, n = 19, vs. 20.7 ± 7.1 ng/ml in untreated subjects, n = 9, p = 0.12) or on montelukast (25.4 ± 10.4 ng/ml, n = 13). Twenty percent of study subjects had uncontrolled asthma (ACT ≤ 19) at baseline (M = 1/F = 5, age 73.3 ± 5.5 years, n = 6). In these subjects, serum vitamin D was significantly lower than in subjects who were well controlled (Table 3).

**Table 3:** **Serum vitamin D at different inhaled steroids doses and ACT scores**

	I.C.S. dose (mcg/day)	Score	Vitamin D (ng/ml)	P value
I.C.S. >400 mcg/day	687 ± 248	-	22.8 ± 9.6	
				0.27
I.C.S. ≤400 mcg/day	134 ± 107	-	26.6 ± 8.5	
ACT ≤19	-	17 ± 1.7	19 ± 5.6	
				0.045
ACT >19	-	23.9 ± 1.3	25.7 ± 9.6	

*I.C.S. = Inhaled corticosteroids.*

In subjects with lower baseline FEV_1%_ (<70% of the predicted value, mean 57 ± 8.9%, n = 13), serum vitamin D was essentially the same as in the rest of the study subjects (23.9 ± 6.7 ng/ml). We found no association between serum vitamin D and baseline FEV_1%_ (r = 0.34, p = 0.08), FEV_1_/FVC (r = 0.11, p = 0.58), or FEF_25-75%_ (r = -0.11, p = 0.58), although there was a trend for an association with FEV_1%_.

**Figure 1:**
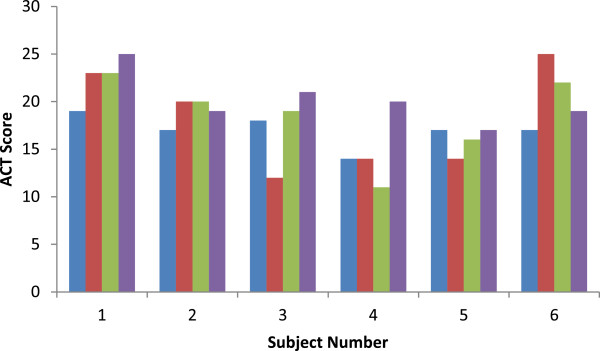
**Effect of vitamin D supplementation on ACT scores in subjects with uncontrolled asthma.** Individual columns (blue, red, green, and purple) represent ACT values obtained at baseline and at 4, 8, and 12 weeks, respectively for the six subjects.

Supplementation of vitamin D3 (2,000 I.U./day) for 12 weeks lead to a significant increase in its serum levels of approximately 10 ng/ml (to 34 ± 7.1 ng/ml, p < 0.001). Overall, FEV_1%_ and ACT scores did not vary throughout the study period (70.5 ± 15, p = 0.99, and 22.8 ± 2.9, p = 0.66 at 12 weeks, respectively). In subjects with uncontrolled asthma (ACT = 17 ± 1.7), ACT scores similarly did not change at 4 and 8 weeks (18 ± 0.7 and 18.5 ± 4.4, respectively, p > 0.46), However, in these subjects ACT scores increased significantly after 12 weeks of vitamin D3 supplementation (20.2 ± 2.7, p = 0.039) (Figure 1). In these subjects, the asthma treatment was essentially unchanged (inhaled steroid dose at baseline = 620 ± 263 mcg/day vs. 718 ± 314 mcg/day at 12 weeks, p = 0.57) and serum vitamin D increased to 32.3 ± 4.7 ng/ml (p = 0.001). In the same subjects with uncontrolled asthma, and in those with baseline FEV_1_ < 70%, FEV_1%_ did not change for the duration of the study (p = 0.79 and p = 0.88 at 12 weeks, respectively). FEV_1_/FVC and FEF_25-75%_ similarly remained unchanged throughout the study period (data not shown).

As expected, vitamin D3 supplementation was well tolerated and none of the study subjects complained of adverse effects from this treatment during the study period. Serum calcium levels remained the same at the end of the study (9.55 ± 0.42 mg/dl). However, in two study subjects, with baseline serum calcium of 10.2 mg/dl and 9.6 mg/dl, these levels increased at 12 weeks to 10.7 mg/dl and 10.5 mg/dl, respectively.

### Discussion

In the present study we investigated the role of vitamin D in a group of elderly subjects with asthma. Similar to our study, it has been recently reported that allergic sensitization is very common in asthmatic patients older than 55 years old [12].

In our study, 79% of the subjects had lower than normal serum vitamin D at baseline. These results confirm in elderly subjects that vitamin D deficiency and insufficiency are extremely common in patients with asthma and respiratory disease [13, 14]. Vitamin D deficiency is more common with obesity, and serum vitamin D levels are inversely related to BMI [10]. We did not find an association between serum vitamin D and BMI, age or duration of asthma, and its values were similar in men and women. Such difference may be explained by the fact that only two of our study subjects had a BMI of 30 or higher.

Serum vitamin D has been found to be inversely, although weakly correlated with inhaled steroid dose in children [13]. Our results failed to show such an association and are in agreement with previous results in asthmatic adults [13]. Serum vitamin D did not vary in subjects treated with long-acting bronchodilators or montelukast.

In children, serum vitamin D was positively associated with the ACT scores [15]. We found that in elderly subjects with uncontrolled asthma (<19) serum vitamin D was significantly lower than in the subjects whose asthma was controlled. Our results are in agreement with a recent study of adult asthmatics that showed that serum vitamin D was lower in subjects with uncontrolled symptoms [16]. We have very recently found that in elderly subjects with uncontrolled symptoms of perennial allergic rhinitis serum vitamin D is inversely related to nasal symptoms (unpublished observations).

FEV_1%_ values have been found positively, albeit weakly associated with serum vitamin D in asthmatic children and adults [15, 17]. In our study, no significant association was found between serum vitamin D and spirometric values, and vitamin D was similar in subjects with lower FEV_1%_ (<70%). It is possible that a larger number of observations would have produced a weak, yet significant association with FEV_1%_ similar to previous studies.

Vitamin D3 supplementation in asthmatic children reduces the risk of recurrent respiratory infections and asthma exacerbations [18]. A small study in mostly non-white children with an average ACT score of 18 failed to show an effect of one year vitamin D supplementation (1,000 I.U./day) on ACT scores and FEV_1%_ [19]. Similarly, we did not find a variation of the spirometric values and ACT scores after a 12 week vitamin D3 supplementation. However, we did find a significant improvement of the ACT scores at the end of the study in the subjects with uncontrolled asthma (ACT < 19). These results may have occurred because of a placebo effect or by chance. However, such findings may encourage studies of vitamin D in uncontrolled elderly asthmatics. A larger study of vitamin D supplementation in adults with asthma with serum vitamin D of less than 30 ng/ml was very recently published [20]. In this study, vitamin D 4,000 I.U./day for 28 weeks did not reduce treatment (inhaled steroid) failure and exacerbations nor did it improve FEV_1_ and asthma control. However, this study differed from ours in that it involved a younger patient population (average age 40 years old), in the obese range (average BMI 32), almost 50% non-white, and enrolled subjects throughout the different seasons [20]. Another very recent study of adult asthmatics reported an association between vitamin D sufficiency and decreased exacerbations and emergency room visits over a five year period [21].

Whereas expectedly vitamin D3 supplementation was well tolerated, we found a mild elevation of the serum calcium in two subjects after 12 weeks. This suggests that in elderly subjects it would be reasonable to measure serum calcium before and after several months of vitamin D supplementation.

Limitations of our study include the relatively small number of subjects, almost exclusively caucasian with well controlled asthma, and the lack of a placebo group. However, the results of our study in elderly asthmatics appear to confirm previous findings about the importance of vitamin D in asthma and may encourage larger studies in this patients group.

### Conclusions

In summary, our pilot study suggests that vitamin D may play a role in elderly asthmatics. Our results will require confirmation in larger patient cohorts.

## Abbreviations

ACT: Asthma control test
BMI: Body mass index.
